# Effects of laboratory domestication on the rodent gut microbiome

**DOI:** 10.1038/s43705-021-00053-9

**Published:** 2021-09-17

**Authors:** Kate L. Bowerman, Sarah C. L. Knowles, Janette E. Bradley, Laima Baltrūnaitė, Michael D. J. Lynch, Kathryn M. Jones, Philip Hugenholtz

**Affiliations:** 1 School of Chemistry and Molecular Biosciences, Australian Centre for Ecogenomics, The University of Queensland, Brisbane, QLD Australia; 2 Department of Zoology, University of Oxford, Oxford, UK; 3 School of Life Sciences, University of Nottingham, Nottingham, UK; 4 Nature Research Centre, Akademijos Str. 2, Vilnius, Lithuania; 5 Department of Biology, University of Waterloo, Waterloo, ON Canada; 6 Department of Biological Science, Florida State University, Tallahassee, FL USA

**Keywords:** Microbiome, Metagenomics

## Abstract

The domestication of the laboratory mouse has influenced the composition of its native gut microbiome, which is now known to differ from that of its wild ancestor. However, limited exploration of the rodent gut microbiome beyond the model species *Mus musculus* has made it difficult to interpret microbiome variation in a broader phylogenetic context. Here, we analyse 120 de novo and 469 public metagenomically-sequenced faecal and caecal samples from 16 rodent hosts representing wild, laboratory and captive lifestyles. Distinct gut bacterial communities were observed between rodent host genera, with broadly distributed species originating from the as-yet-uncultured bacterial genera *UBA9475* and *UBA2821* in the families *Oscillospiraceae* and *Lachnospiraceae*, respectively. In laboratory mice, *Helicobacteraceae* were generally depleted relative to wild mice and specific *Muribaculaceae* populations were enriched in different laboratory facilities, suggesting facility-specific outgrowths of this historically dominant rodent gut family. Several bacterial families of clinical interest, including *Akkermansiaceae*, *Streptococcaceae* and *Enterobacteriaceae*, were inferred to have gained over half of their representative species in mice within the laboratory environment, being undetected in most wild rodents and suggesting an association between laboratory domestication and pathobiont emergence.

## Introduction

The process of animal domestication involves significant changes to the environment, diet and genetics of the organism. Domesticated animals have been demonstrated to harbour a gut microbiome distinct from their wild relatives [1–4], however the consequences of this departure from the native gut community are not fully understood. Laboratory mice form the basis of much of our clinically-oriented understanding of the gut microbiome, yet the first inbred line of laboratory mice was only established in 1909 [5], making the model a very recent derivative of its wild ancestors and one that has now also been demonstrated to host a distinct gut microbiome from that of its wild contemporaries [6, 7].

Initial comparisons of wild and laboratory mice focused on the immune system, demonstrating wild mice are in a generally higher immune activation state than laboratory mice [8, 9]. The laboratory mouse immune system displays characteristics of human neonates, while wild and pet store mice more closely resemble that of human adults [9]. Cohousing laboratory mice with pet store mice significantly altered the composition of their innate and adaptive immune systems, increasing the resistance of laboratory mice to infection with *Listeria monocytogenes*, consistent with the gut microbiome contributing to immune status [9]. Subsequent studies demonstrated that the process of rewilding laboratory mice with their native gut microbiome can improve host fitness. Reconstitution of laboratory mice with a wild mouse microbiome via oral gavage of pregnant females produced offspring largely resistant to infection with mouse-adapted influenza A and to colitis-associated colorectal cancer [6]. Reconstitution via implantation of laboratory mouse embryos into wild mice produced offspring displaying immune responses more closely resembling that of adult humans [7]. Rewilding via introduction of laboratory mice to a controlled outdoor environment resulted in improved maturation and activation of the immune system associated with colonisation by fungi [10]. These data demonstrate that the observed differences between the gut microbiomes of laboratory and wild mice are phenotypically relevant, prompting discussion of the inclusion of ‘dirty’ mice within the model system [11].

Introduction to the laboratory environment has been associated with significant alteration of the gut microbiome of wild *Mus musculus*, with increased abundance of members of the bacterial families *Rikenellaceae* and *Bacteroidaceae* observed in wild-derived colony progeny (F2 generation) in comparison with wild mice, along with decreased abundance of members of the families *Lachnospiraceae*, *Lactobacillaceae*, *Ruminococcaceae* and *Porphyromonadaceae* [12]. Comparison of caecal and ileocecal samples from wild and established laboratory model mice (C57BL/6) revealed wild mice contain a higher abundance of *Bacteroidetes* and *Proteobacteria* and a lower abundance of *Firmicutes* [6, 7]. However different strains of laboratory mice, as well as the same strain from different providers, often harbour different microbial communities [13]. Similarly, variation within the gut microbiome of wild *Mus musculus* has been observed and attributed both to geographic origin [14, 15] and to the level of consumption of plant-derived food sources [16]. Therefore, further exploration of the distinction between wild and laboratory mice is needed. Using a combination of new and previously sequenced metagenomic datasets, we describe genome-centric taxonomic and functional characteristics of both faecal and caecal bacterial communities discriminatory between laboratory and wild mice. We also provide contextual understanding of the observed distinction between the two cohorts by extending our analysis beyond *Mus musculus domesticus* to an additional 15 rodent species. Comparison of related rodent species enabled inference of bacterial gain/loss events within the laboratory environment including several species of clinical interest. We also undertook a cross-host comparison of the recently described gut bacterial family *Muribaculaceae* (formerly *S24-7*; [17]), revealing that the dominance of this family in laboratory mice is not abnormal within rodents, however, *Muribaculaceae* species abundance may be artificially inflated on a laboratory-specific basis.

## Materials and methods

### Sequencing, assembly and binning

Distal colon, caecal and faecal samples from wild *Apodemus agrarius* (striped field mouse), *Apodemus flavicollis* (yellow-necked field mouse), *Microtus agrestis* (field vole), *Microtus arvalis* (common vole), *Microtus oeconomus* (root vole), *Myodes glareolus* (bank vole), and faecal samples from *Mus musculus domesticus* were collected as previously described [18, 19]. DNA was extracted using the PowerSoil DNA Isolation Kit (QIAGEN, Hilden, Germany). Sequencing libraries were generated using the Illumina Nextera DNA Flex kit and sequenced on the Illumina NextSeq 500 platform generating an average of 6 Gbp of 151 bp paired-end reads per sample. Public datasets were downloaded from the NCBI Sequence Read Archive and MG-RAST as indicated (Supplementary Table 1).

Reads from all datasets were cleaned of adapters and quality trimmed using Trimmomatic v0.39 [20] with the following settings: SLIDINGWINDOW:4:15 LEADING:3 TRAILING:3 MINLEN:50. Reads aligning to the host genome were removed using CoverM v0.4.0 (https://github.com/wwood/CoverM) ‘contig’ to map to the relevant genome (--mapper “bwa-mem”) and “filter” to collect unmapped reads. Host reads were identified using a minimum read alignment percentage of 70% for all samples and a minimum percent identity of either 95% where a reference genome of the same species was available or 90% where only a genus level match was available (Supplementary Table 1).

Datasets with average sequencing depth >1 Gbp were assembled using SPAdes v3.13.0/v3.14.0 [21] (--meta -k 21,33,55,77) or MEGAHIT v1.2.9 [22] (--presets meta-sensitive). Publicly available projects with lower sequencing depth were co-assembled per sample type per host using MEGAHIT v1.1.3/v1.2.9 [22]. Method and assembler are specified in Supplementary Table 2. CoverM v0.4.0 “make” was used to map reads to the resulting assemblies (default settings) and samples were then binned using MetaBAT v2.12.1 [23], MaxBin v2.2.4 [24] and CONCOCT v1.1.0 [25]. UniteM v0.0.15 (https://github.com/dparks1134/UniteM) was used to compare bins and identify a non-redundant set. RefineM v0.0.24 (https://github.com/dparks1134/RefineM) was used to identify and filter potential contaminant contigs based on GC content, tetranucleotide frequency and taxon assignment. Bin quality metrics were calculated using CheckM v1.1.2 [26] and taxonomy was assigned using GTDB-Tk v1.3.0 [27–29] using releases 05-RS95 and 06-RS202. Isolate samples from the mouse intestinal bacterial collection (miBC) [30] were assembled de novo using CLC Assembly Cell v4.4 (-m 500) (QIAGEN, Hilden, Germany). Genomes were annotated using DRAM v1.0.6 [31].

### Genome database

To create a species level genome database, recovered MAGs >50% complete with <5% contamination were collated and combined with 1,296 species representative MAGs from the integrated mouse gut metagenomic catalogue (iMGMC) [13] and 63 isolate genomes from the miBC [30] before dereplication at 95% using dRep v2.5.4 [32] with an alignment fraction of 0.65 (--SkipMash --S_algorithm fastANI -sa 0.95 -nc 0.65). Dereplicated genomes were combined with the 24,627 non-overlapping bacterial genomes contained in GTDB release 04-RS89. Where GTDB-Tk indicated a MAG was within the GTDB species cluster of a published MAG, the more complete MAG was retained. Where the species cluster was represented by an isolate genome, the isolate genome was retained. Mapping counts to the final database of 30,633 concatenated genomes (Supplementary Table 3) were obtained using CoverM (--mapper “bwa-mem” --min-read-aligned-percent 70 --min-read-percent-identity 0.95). Unmapped reads were collected using CoverM and mapped to the subsequently released GTDB 05-RS95 (new species representatives only, 7580 genomes) and 108 mouse isolate genomes from the mouse gut microbial biobank (mGMB) [33] (Supplementary Table 3). Eleven genomes from the mGMB were excluded based on GTDB-Tk warnings of multiple markers with multiple hits plus potential mixed domain origin.

Relative abundance of each MAG was calculated based on reads mapped per genome scaled to account for genome size (total nucleotide length of database/genome length). Samples from humans (NCBI BioProjects PRJEB17632 [34] and PRJNA278393 [35]) and pigs (NCBI BioProjects PRJEB11755 [36] and PRJNA352989 [37]) were included as outgroup comparisons.

Comparison between metagenome-based and 16S rRNA gene-based relative abundance was undertaken using data from wild rodents retrieved from NCBI BioProject PRJEB30121. Project details have been published previously [18]. Reads were cleaned of adapter sequences using Cutadapt v1.1 [38] and trimmed using Trimmomatic v0.36 [39] employing a sliding window of 4 bases with an average base quality above 15, followed by hard-trimming to 250 bases with exclusion of reads less than this length. Remaining forward reads were processed following the QIIME2 workflow [40] using DADA2 v1.12 [41] to denoise sequences. Taxonomy assignment was performed on amplicon sequence variants using BLAST v2.8.1 [42] against the SILVA [43] reference database version 132.

Alpha diversity metrics (Shannon and Simpson) were calculated based on genome size scaled mapping counts with the “estimate_richness” function within the R package phyloseq v1.34.0 [44]. Aitchison distance (Euclidean distance from centred log ratio transformed mapping counts) was calculated using the base R v4.0.3 “dist” function. Global comparison of alpha diversity and Aitchison distance across all rodent hosts was undertaken using the Kruskal-Wallis test implemented within the base R v4.0.3 “kruskal.test” function. Subsequent pair-wise inter-host comparison was performed using Dunn’s multiple comparison test implemented in the R package dunn.test v1.3.5 [45] with Benjamini-Hochberg adjustment. Rodent hosts represented by ≤4 samples were excluded from diversity analysis.

### Bacterial tree

Genomes from the species level genome database described above recruiting ≥500 reads in ≥1 sample with covered fraction of ≥0.01 were included in the bacterial tree (*n* = 9411). Multi-sequence alignment was generated using GTDB-Tk v1.3.0 [27] and maximum likelihood tree inferred using IQ-TREE v1.6.12 [46] using ModelFinder for model selection across LG model set (-m MFP -mset LG -bb 1000). Bootstrap support was generated from 1,000 ultrafast bootstrapping replicates, and the tree was visualised using iTOL [47].

Mean nearest taxon distance and mean pairwise distance were calculated using the “ses.mntd” and “ses.mpd” functions within the R package picante v1.8.2 [48]. Significance is determined within the functions based on deviation from an independent swap null model maintaining species occurrence frequency and sample species richness with mean calculated from 1000 iterations. A phylogenetic distance matrix was calculated using the “cophenetic” function within base R v4.0.3.

### Rodent tree

The host phylogenetic tree was inferred using four nuclear (*IRBP*, *GHR*, *BRCA1*, *RAG1*) and two mitochondrial genes (12S rRNA, *CYTB*), which were selected based on sequence availability and previous rodent phylogenetic studies [49, 50]. Nucleotide sequences used are contained in Supplementary Table 4. Sequences were aligned using MAFFT v7.455 [51] and trimmed of gaps present in >10% of sequences using trimAl v1.4.1 [52]. Maximum likelihood trees were inferred using IQ-TREE v1.6.12 [46] using ModelFinder for model selection with gene and codon partitioning (options: -m MFP -bb 10000). Bootstrap support was generated from 10,000 ultrafast bootstrapping replicates.

### Comparison of community composition and genome-based functional profiles

Comparison of the faecal and caecal bacterial communities was performed using ALDEx2 v1.18.0 [53] and/or mixOmics v6.14.0 [54] using CLR-transformed read mapping counts for genomes passing the presence threshold of ≥500 reads mapped with coverage fraction of ≥0.01. MixOmics-implemented-sPLS-DA was run using parameters determined by the “tune.splsda” function with 10 × 50-fold cross-validation. ALDEx2 was run using 1000 Monte Carlo samples and Wilcoxon signed-rank test of significance. Analysis included faecal or caecal samples only and excluded laboratory mice subject to some form of treatment.

Comparison of functional annotation of genomes was performed using DRAM generated KEGG, CAZy and Pfam annotations with groups defined as genomes enriched in particular rodent hosts. Annotations were filtered for those present in the equivalent of 10% of the smaller comparison group or as indicated in figure legends. Analysis undertaken using ALDEx2 and/or mixOmics as described above.

### Inference of gain/loss events

Bacterial species gain/loss events in each host were inferred based on presence/absence matrices generated from faecal or caecal samples using command-line implementation of Count [55] at the bacterial family level. Likelihood rates were optimised using the “ML” function (-max_paralogs 1000 -opt_rounds 100 -gain_k 1 -loss_k 1 -length_k 1) and gain/loss events inferred using posterior probability (“Posteriors” function). Events were filtered for those with probability ≥80%.

### Additional statistical analyses

PCA was performed using the “rda” function within the vegan v2.5-5 R package [56]. PCA plots and bar graphs were generated using the ggplot2 v3.3.2 R package [57].

## Results & discussion

**Fig. 1:**
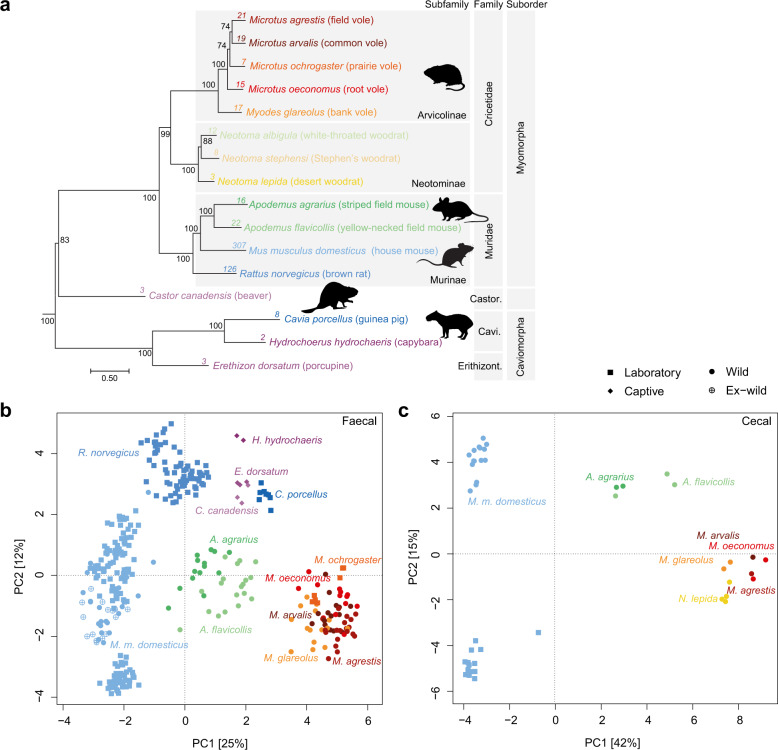
The rodent gut microbiome is distinguishable by host genus. **a** Maximum likelihood tree of rodent hosts included in the dataset based on alignment of two mitochondrial and four nuclear genes, as described in Supplementary Table 4. Bootstrap support generated from 10,000 ultrafast bootstrapping replicates shown on interior nodes. Sample numbers per host indicated at tips. PCA of (**b**) faecal and (**c**) caecal samples based on read mapping counts (CLR-transformed) to genome database filtered to include genomes recruiting ≥500 reads across ≥0.01 of the genome in ≥1 sample. Ex-wild samples represent animals transferred from the wild to the laboratory.

To extend our understanding of the rodent gut microbiome beyond the laboratory mouse we examined a combination of publicly available and new metagenomic sequencing data (Supplementary Tables 1 and 2). We identified public data for nine additional rodent species from wild, captive (e.g., wildlife parks) and laboratory sources: *Rattus norvegicus* (brown rat, laboratory samples [58–60]), *Microtus ochrogaster* (prairie vole, laboratory samples [61]), *Neotoma albigula* (white-throated woodrat, wild samples [62]), *Neotoma stephensi* (Stephen’s woodrat, wild samples [62]), *Neotoma lepida* (desert woodrat, laboratory samples [63], recently captured), *Castor canadensis* (beaver, captive samples [64]), *Erethizon dorsatum* (porcupine, captive samples [64]), *Cavia porcellus* (guinea pig, laboratory samples [65]) and *Hydrochoerus hydrochaeris* (capybara, captive samples) (Fig. 1a). To these hosts we added samples from wild rodent populations obtained in Lithuania; *Apodemus agrarius* (striped field mouse), *Apodemus flavicollis* (yellow-necked field mouse), *Microtus agrestis* (field vole), *Microtus arvalis* (common vole), *Microtus oeconomus* (root vole), *Myodes glareolus* (bank vole), and wild *Mus musculus domesticus* samples obtained from the Isle of May, UK. Additional wild *M. m. domesticus* samples were obtained from three publicly available datasets [6, 7, 16]. For comparison, laboratory *M. m. domesticus* representatives were included from these same studies in addition to those from a large study of multiple laboratory mouse strains [66]. No gene deletion mouse strains were included in our study to remove the additional variable of gut microbiome alterations introduced via genetic manipulation of the host.

Metagenome assembled genomes (MAGs) were recovered from samples on a per-sample basis, with the exception of five co-assemblies of low sequencing depth samples from the same host species and location. Combining these MAGs with publicly available reference genomes, we compiled a genome database that recruited up to 92% (average 74%) of sample reads (Supplementary Fig. 1b) producing community profiles that discriminate between host genera (Fig. 1b, c). A clear distinction in mapping rates was evident between the laboratory models (*M. m. domesticus* and *R. norvegicus*) and most other rodent hosts reflecting the current database bias toward model species (Supplementary Fig. 1). Despite the lower read mapping to non-model host genera, the compiled reference genomes appear to be representative of the gut microbiomes of the Lithuanian wild rodent cohort previously analysed with 16S rRNA gene amplicon sequencing [18]. For example, the relative abundance of the three most dominant bacterial families in the Lithuanian samples was consistent between the two methods despite the metagenomic profiles being based on mapped reads only, suggesting that there were no substantial gaps in bacterial diversity estimates (Supplementary Fig. 2).

### Bacterial species distribution in the gut microbiomes of sixteen rodent hosts

**Fig. 2:**
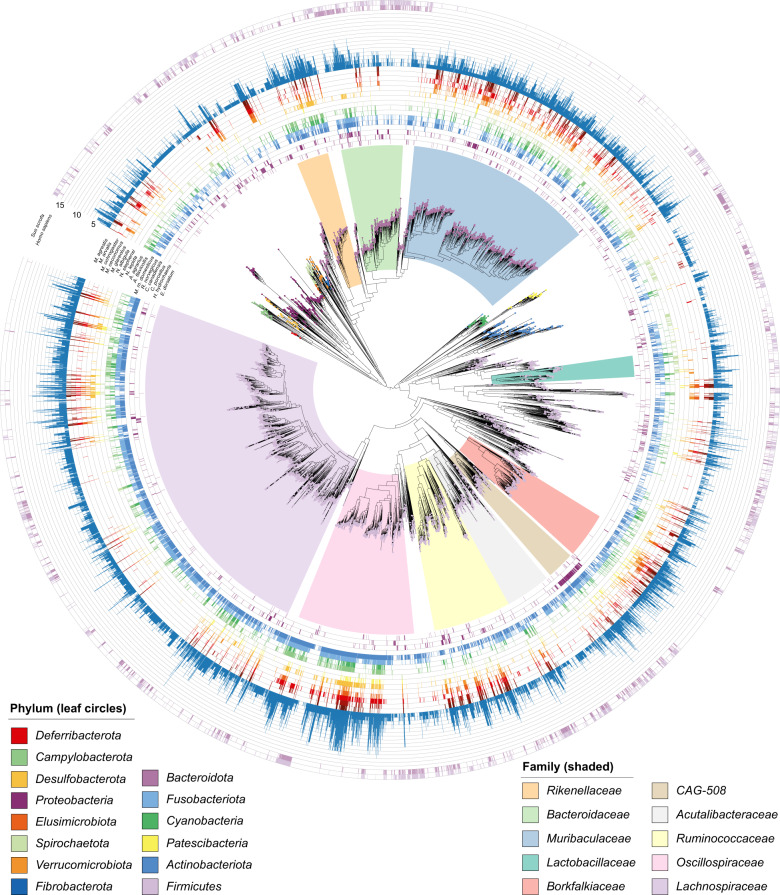
Bacterial species detection across the rodent gut microbiome. Maximum likelihood tree inferred based on alignment of 120 single copy marker genes identified by GTDB-Tk [27] within genomes recruiting ≥500 reads across ≥0.01 of the genome in ≥1 sample (9411). Outer rings show presence of each bacterial species in a given rodent host where presence is based on meeting the same recruitment criteria in at least one sample per rodent host (16 rings). Blue bar graph displays the number of rodent hosts meeting this threshold for each bacterial species, i.e., rodent host range. Outer two rings display presence in outgroup species, *Homo sapiens* and *Sus scrofa* (pig). Coloured circle on leaf indicates phylum. Coloured ranges indicate families with relative abundance ≥0.5% in >300 samples.

**Fig. 3:**
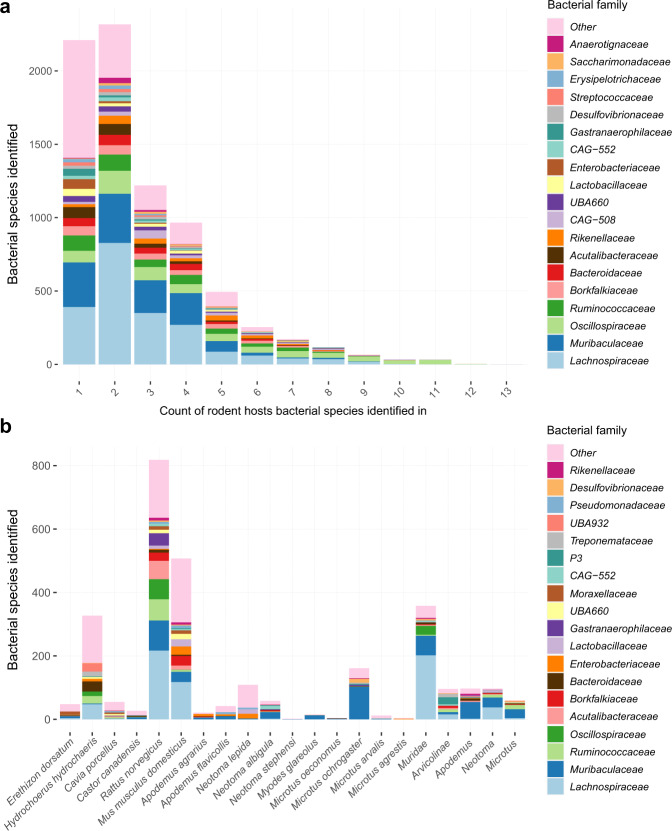
Distribution of bacterial species across rodent hosts. **a** Count of rodent hosts each bacterial species was identified in based on all gut microbiome samples within the dataset. **b** Count of bacterial species identified exclusively within either a single rodent host, genus or family. For rodent genera and families, bacterial species were included only where they were not identified in samples outside of the specified group. Presence of bacterial species within a given rodent host species based on genomes recruiting ≥500 reads across ≥0.01 of the genome in ≥1 sample.

The most widely distributed bacterial species across the sampled rodents was an unnamed member of the genus *UBA2821* within the family *Lachnospiraceae*, present in 13 of the 16 hosts (Fig. 2, Supplementary Table 5). Most other broadly distributed species (present in ≥10 hosts, *n* = 70) were members of the family *Oscillospiraceae* (58 species, Fig. 3a). The majority of these species (54/58) were not identified in human and pig faecal samples used as phylogenetic outgroups. Within Murinae hosts (Fig. 1a), 56% of the bacterial species identified exclusively in all four representatives belong to the family *Lachnospiraceae* (202/358 species) and 17% to the family *Muribaculaceae* (62/358 species, Fig. 3b, Supplementary Table 6). Within Arvicolinae hosts (voles), 23% of species identified exclusively within this host group were from the family *P3* (22/96, order *Bacteroidales*) and 17% from the family *Lachnospiraceae* (16/96, Supplementary Table 6). At host genus level, 97 bacterial species were exclusively identified in all *Apodemus* hosts, 60 in *Microtus* hosts and 97 in *Neotoma* hosts, indicating potential genus-level specificity (Supplementary Table 6). Members of the families *Lachnospiraceae* and *Muribaculaceae* were the most represented in each of these host genus-level exclusive groups (Fig. 3b). Over 2,200 bacterial species were identified in a single host species, of which 507 and 818 were present in *M. m. domesticus* and *R. norvegicus* respectively, representing 12% and 17% of the total species identified in each of these hosts and reflecting the substantially higher sample number and sequencing depth for these rodents compared to other host species (Fig. 3b). As with species identified exclusively within higher rodent taxonomic groups (genus, family), members of the family *Lachnospiraceae* and *Muribaculaceae* comprised the majority of bacterial species identified within a single rodent host species (Supplementary Table 6).

### Comparison of laboratory and wild mice

**Fig. 4:**
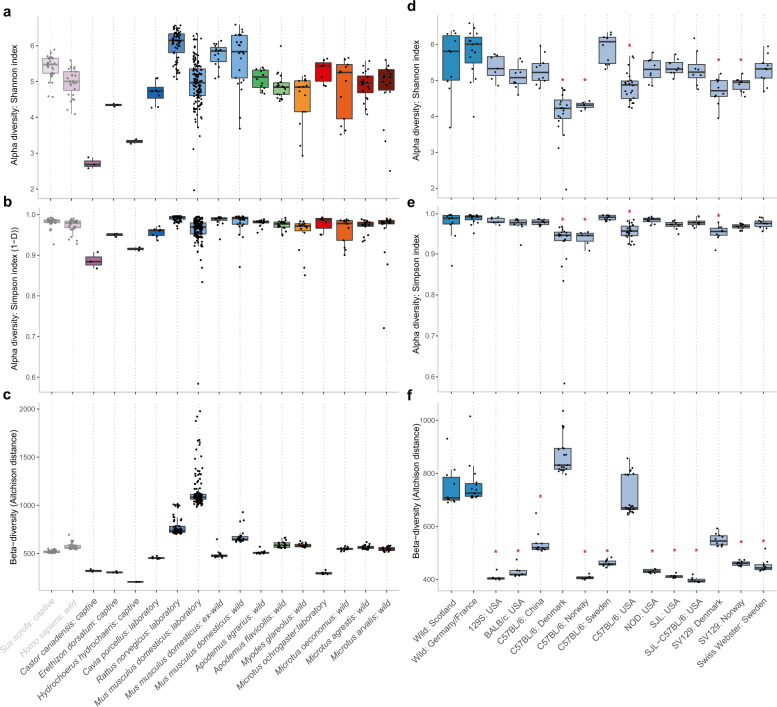
Diversity metrics across rodent faecal samples. Alpha diversity measured using (**a**), (**d**) Shannon and (**b**), (**e**) Simpson (1-D) indices based on genome size scaled mapping counts. Global analysis undertaken using Kruskal-Wallis test (*p* < 0.0001). **c**, **f** Beta diversity measured using Aitchison distance (Euclidean distance from CLR transformed mapping counts). Global analysis undertaken using Kruskal-Wallis test (*p* < 0.0001). Pairwise significance determined using Dunn’s multiple comparison test with Benjamini-Hochberg adjustment—see Supplementary Tables 7 and 8. **d–f** Red asterisk indicates significant difference of laboratory cohort from both wild mouse groups. Analysis includes untreated faecal samples only. Hosts represented by <5 samples excluded (*Castor canadensis*, *Erethizon dorsatum*, *Hydrochoerus hydrochaeris*).

Alpha diversity was significantly greater in wild *M. m. domesticus* faecal samples in comparison to other wild hosts, which may also reflect database bias toward this species (Fig. 4a, b, Supplementary Table 7, analysis includes untreated faecal samples only and hosts represented by ≥5 samples). Alpha diversity within laboratory mice was significantly lower than that of wild mice, likely in part due to dietary changes. Wild mice fed a standard laboratory chow have been shown to experience a reduction in diversity over time, and, conversely, laboratory mice display increased diversity when fed a wild diet [4]. In contrast, variation among laboratory mouse samples (beta diversity) was significantly higher compared to wild mice (Fig. 4c, Supplementary Table 7). The high inter-sample variation between laboratory mice reflects the inclusion of multiple laboratory mouse datasets and the known dependence of the laboratory mouse gut microbiome on variables such as animal strain, host laboratory and diet [reviewed in [67]]. Comparison at the level of individual laboratory mouse cohorts confirmed distinction from wild mice varied between cohorts (Fig. 4d–f, Supplementary Table 8). While only a subset (5/13) of groups displayed significantly reduced alpha diversity in comparison to wild mice, beta diversity was reduced in the majority (10/13), in agreement with defined conditions within an individual laboratory constraining variability within that facility.

**Fig. 5:**
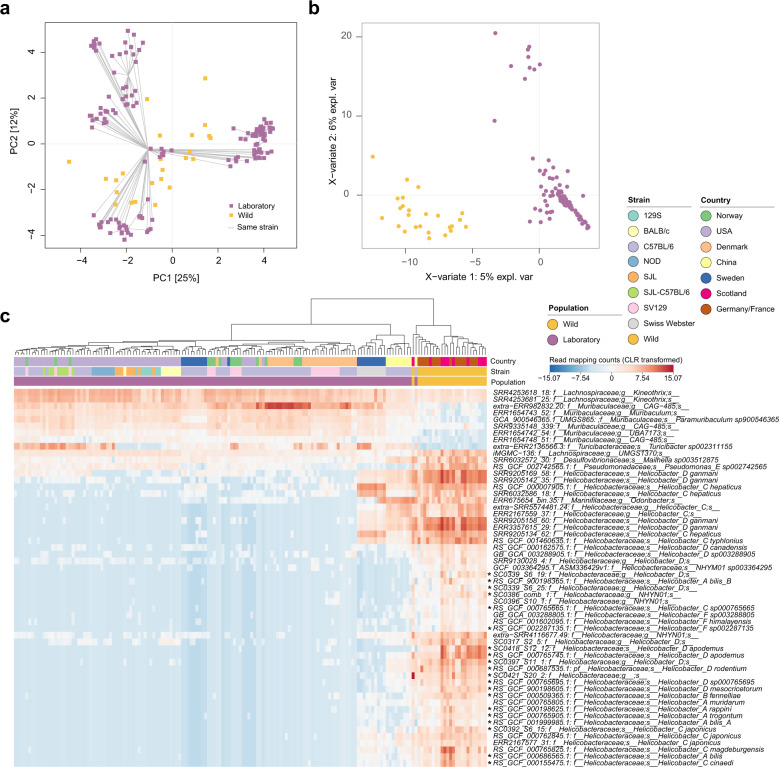
Bacterial species distinguishing the laboratory mouse faecal microbiome from that of wild mice. Analysis undertaken using faecal samples from laboratory mice fed a standard chow diet and wild mice. **a** PCA and (**b**) sPLS-DA based on read mapping counts (CLR-transformed) to genome database filtered to include genomes recruiting ≥500 reads across ≥0.01 of the genome in ≥1 sample. **c** Heatmap containing genomes identified as discriminatory between laboratory and wild mice using ALDEx2 (effect ≥ |1.5 | ) and sPLS-DA implemented within mixOmics. Genomes selected by both methods marked with an asterisk, remainder identified with ALDEx2 only.

Comparison of the faecal bacterial community between wild and laboratory mice identified *Helicobacter* species as the predominant enriched taxa in wild mice and members of the family *Muribaculaceae* as predominant in laboratory mice (Fig. 5, Supplementary Table 9, includes standard chow-fed laboratory mice only), as recently observed [68]. *Helicobacter* are known colonisers of rodents and their presence in laboratory mice is closely monitored due to their ability to cause inflammation in some inbred mouse lines influencing disease outcomes [reviewed in [67]]. In contrast, wild mice experience exposure to microorganisms considered pathogenic in the laboratory mouse, such as *Helicobacter* spp. without showing signs of disease [6]. Only three of the 14 laboratory mouse cohorts examined contained *Helicobacter* spp., consistent with eradication on a laboratory/colony basis (Supplementary Table 10). Genome-centric functional comparison of the microbial species enriched in either laboratory or wild mice revealed partial separation between the two groups driven primarily by features encoded in members of the families *Muribaculaceae* and *Helicobacteraceae* (Supplementary Fig. 3, Supplementary Tables 11-13). For example, several glycoside hydrolases, including α- and β-glucosidases (GH13, GH31 and GH3 (β)), β-galactosidases (GH2) and β-xylosidases (GH43), as well as components of the starch-utilization system, were enriched in laboratory mice, suggesting diet as a potential driver of enrichment. Increased abundance of glycoside hydrolases, including GH31, in domesticated animals has been observed previously, hypothesised to be associated with an altered variety of plant carbohydrates in commercial feeds [3]. Multiple glycosyltransferases involved in lipopolysaccharide and peptidoglycan biosynthesis were enriched in wild mice, potentially contributing to an increased range of glycosylation patterns that could prove inflammatory in the laboratory mouse [69]. Flagellar and chemotaxis protein domains (including basal body components and flagellin) were also enriched amongst the wild mouse associated species. Motility related genes were observed as enriched within one of two enterotypes previously described in wild mice (samples from PRJEB32890, Supplementary Table 2) [16] suggesting differential enrichment of motility across wild mice. Within the laboratory environment, flagellin administration has been demonstrated as protective against induced colitis via induction of anti-flagellin IgA and microbiome modulation [70]. The reduction of native mouse flagellated species within laboratory mice may therefore contribute to their altered immune status [8].

### Comparison of laboratory and wild voles

As a means of supporting the identified distinction between wild and laboratory mouse samples we undertook a similar comparison of wild and laboratory voles, comparing the three wild sampled *Microtus* voles, *M. arvalis*, *M. agrestis* and *M. oeconomus*, to the laboratory model *M. ochrogaster*. Over 1700 species were identified as enriched in either the wild (961 species) or laboratory (826 species) *Microtus* hosts, substantially more than the set of species that distinguish laboratory and wild *M. m. domesticus* samples (Supplementary Table 14). The increased variation is likely due in part to the comparison of different *Microtus* species as opposed to *M. m. domesticus* strains, as well as sampling differences owing to the two groups originating from different projects; colon contents were obtained for the wild hosts vs faecal samples for the laboratory host. Despite these confounding factors, 37 species enriched in wild or laboratory *Microtus* hosts shared the same enrichment pattern between wild and laboratory mice (29% of the species identified as discriminatory in mice), the bulk of which comprise members of the families *Helicobacteraceae* and *Muribaculaceae* (Supplementary Table 14). Increased family-level abundance of *Muribaculaceae* and decreased *Helicobacteraceae* was also observed by 16S rRNA amplicon profiling in newly captive populations of deer mice (without active control) suggesting that certain *Muribaculaceae* species bloom with exposure to laboratory diets while *Helicobacteraceae* species may require continual reintroduction from the environment to maintain high colonisation level [71]. In contrast to the laboratory vs wild mice analysis, laboratory and wild voles were less distinct based on multivariate comparison of the functional repertoires of the differential genomes (Supplementary Fig. 4). Alternate microbial species are therefore likely performing equivalent functions across the different vole hosts given the observed microbial community divergence between them.

### Extended comparison of laboratory and wild hosts

To examine broader trends amongst laboratory and wild rodents we undertook a comparative faecal community analysis across all Murinae hosts as well as across the whole dataset (Fig. 1a). The majority of microbial species identified as differential in both comparisons were depleted in laboratory mice; 88% amongst Murinae hosts and 91% amongst all hosts (79 & 201 differential species respectively, Supplementary Fig. 5, Supplementary Tables 15 & 16). Similar to the comparison of wild and laboratory mice, we observed depletion of *Helicobacteraceae* in laboratory hosts in both broader comparisons. By contrast, the abundance of multiple members of the family *Muribaculaceae* was depleted in laboratory hosts, however the depleted species differed from those previously identified as enriched specifically in laboratory mice. Other species depleted in laboratory hosts include members of the *Bacteroidales* genera *Odoribacter* and *Alistipes* and *Christensenellales* genera *UBA11940* (family *Borkfalkiaceae*) and *RACS-045* (family *UBA3700*). Comparison of functional annotation between genomes enriched in each group identified increased numbers of spore formation and cell envelope-associated domains within laboratory enriched species (Supplementary Fig. 6, Supplementary Tables 17 and 18). These annotations were encoded within genomes from a number of species including *Turicibacter sp002311155*, *Kineothrix* sp., *Harryflintia acetispora*, and members of a number of as-yet-uncultured genera including *HGM13010* and *CAG-110* (family *Oscillospiraceae*) and *QAMM01* and *UBA3818* (family *Ruminococcaceae*). Each of these species were either not identified or found at low prevalence in wild rodent hosts (Supplementary Tables 15 and 16). Spore formation is common in human gut associated species [72] and could represent a means of transfer in the laboratory environment, however, due to the small number of laboratory enriched species, the possibility of an artificial signal cannot be excluded.

Comparison of caecal samples from wild hosts vs those from laboratory mice (samples from Fig. 1c) revealed greater distinction, with over 1500 species distinguishing the two groups; 371 enriched in wild hosts and 1162 in laboratory mice (Supplementary Table 19). The majority (87%) of these species remained distinct when wild mouse samples were excluded from the comparison. Discriminatory species typically belonged to bacterial families that were enriched in either laboratory or wild samples. For example, 542 members of the family *Lachnospiraceae* were enriched in laboratory mice vs nine in wild hosts while 185 members of the family *Muribaculaceae* were enriched in wild hosts vs nine in laboratory mice (Supplementary Table 19). Functional comparison of genomes enriched in either wild or laboratory rodents identified a number of cell envelope-associated factors as discriminatory between the groups including components of the TonB transport and lipopolysaccharide export systems in wild-associated species and peptidoglycan teichoic acid transferase in laboratory-associated species, suggestive of differences in cell envelope structure between the groups (Supplementary Fig. 7, Supplementary Tables 20–22). Several flagellar proteins were also enriched amongst bacterial species enriched in caecal samples from laboratory mice, in contrast to the observed depletion in faecal laboratory-associated species (Supplementary Tables 12 and 13). In total, 90% of the species enriched in laboratory mice were found to carry genes associated with Gram-positive cell envelopes while 74% of species depleted in laboratory mice encoded genes associated with Gram-negative cell envelopes (Supplementary Table 23) [73]. The dominance of Gram-positive species amongst those enriched in the laboratory mouse caecum is consistent with an overall enrichment of Gram-positive species in that environment; a finding that also extends to laboratory mouse faecal samples (Supplementary Fig. 8a, Supplementary Table 23). Comparison across other rodents, including other laboratory model hosts, confirmed the inflated Gram-positive ratio was only evident in mice (Supplementary Fig. 8a), and additionally, only evident in some laboratories (Supplementary Fig. 8b). Similarly, analysis of laboratory mouse caecal samples from two additional projects [74, 75] confirmed the expansion of Gram-positive species within the caecum of the laboratory mouse appears to be laboratory specific (Supplementary Fig. 8a). This observation is relevant in the context of clinical studies where depletion of Gram-positive species is associated with improved outcomes [76, 77] and suggests this benefit may be connected with the restoration of a microbial community more closely resembling that of the wild ancestors of laboratory mice.

### Presence/absence-based comparison of laboratory and wild rodents

**Fig. 6:**
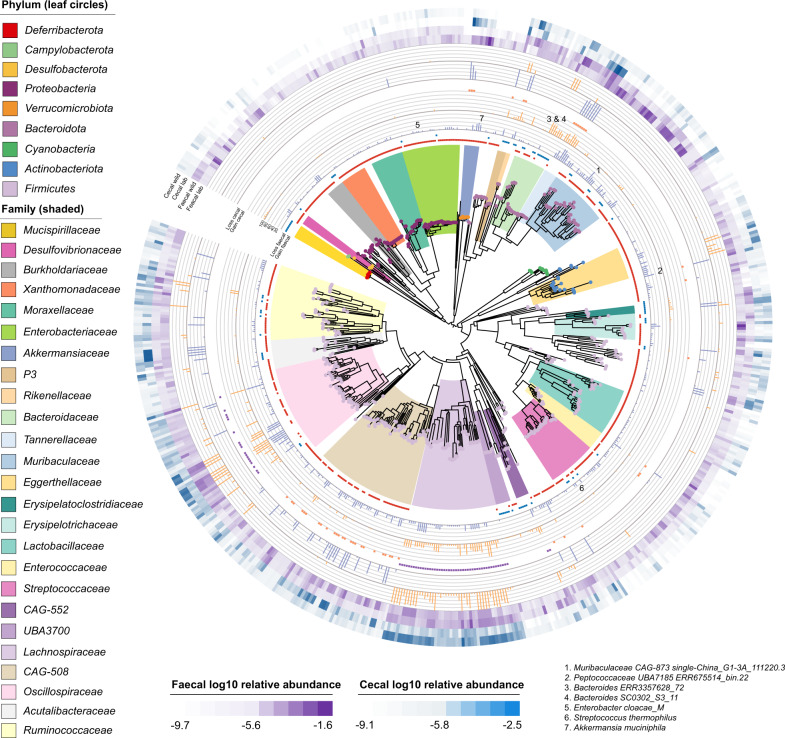
Putative gain/loss events within the laboratory mouse gut microbiome. Tree from Fig. 1 pruned to include only genomes predicted as gained or lost species within the laboratory mouse. Innermost rings indicate gain (red) and loss (blue) events within faecal samples. First two bar charts indicate prevalence of species within faecal samples of laboratory (purple) or wild (orange) mice. Subsequent rings indicate gain (orange) and loss (purple) events within caecal samples and final two bar charts indicate prevalence of species within caecal samples of laboratory (purple) or wild (orange) mice. Heatmaps display log_10_ relative abundance of species within laboratory (purple) or wild (blue) mice. Coloured circle on leaf indicates phylum. Coloured ranges indicate families.

To complement our assessment of enrichment between laboratory and wild rodents we also identified potential gain and loss of species events associated with laboratory usage based on the pattern of presence/absence (putative absence, indistinguishable from species that are below detection) across closely related rodent hosts. Analysis of domesticated hosts has revealed both gain and loss of gut bacterial species following domestication, where gain in some instances is posited to represent transfer from humans [3, 78]. In this dataset, approximately five times more gut microbial species were inferred as gained (452) than lost (82) in the laboratory mouse based on faecal samples since diverging from its wild ancestor (Supplementary Table 24). 87% of putatively gained bacterial species in the laboratory mouse were absent in all wild rodent faecal samples supporting gain as a result of domestication. By comparison, 62% of the species inferred as lost in the laboratory environment were present in at least one wild rodent host beyond *M. m. domesticus*. All species inferred as gained/lost were typically present at low abundance in both wild and laboratory *M. m. domesticus* hosts (<3% mean relative abundance per group), however some displayed high prevalence in either laboratory (e.g., *Muribaculaceae CAG-873 MAG single-China_G1-3A_111220.3* (65%) and *Peptococcaceae UBA7185 MAG ERR675514_bin.22* (52%)) or wild (e.g., *Bacteroides MAGs ERR3357628_72* (56%) and *SC0302_S3_11* (52%)) samples (Fig. 6, Supplementary Table 24). Twenty-two bacterial families were inferred to have gained at least 50% of their representative species in the laboratory environment (excluding singleton families represented by a single bacterial species) including several of clinical interest, e.g., *Moraxellaceae* (18 of 18 species gained), *Xanthomonadaceae* (15 of 15 species gained), *Enterococcaceae* (9 of 9 species gained), *Akkermansiaceae* (8 of 8 species gained), *Streptococcaceae* (23 of 31 species gained) and *Enterobacteriaceae* (31 of 43 species gained) (Fig. 6, Supplementary Table 25). While these species were present at low abundance in the laboratory mice included in this study, their presence is of interest given their potential as opportunistic pathogens. Previous isolation of some of these species from humans (e.g., *Enterobacter cloacae_M*, *Streptococcus thermophilus*, *Akkermansia muciniphila* and *A. muciniphila_A* & _*B*), also supports potential transfer within the laboratory (Supplementary Table 24). Two families were inferred to have lost over 50% of their representative species in the laboratory environment: the uncultured *Bacteroidales* family *P3* (4 of 6 species lost) and *Mucispirillaceae* (7 of 13 species lost) (Supplementary Table 25).

Within caecal samples, 47 species were inferred as gained and 67 lost in the laboratory mouse (Fig. 6, Supplementary Table 24). In total, 95% of the species gained were below detection in all wild rodent hosts, similar to the proportion observed in faecal samples. 19 species were inferred as gained in both faecal and caecal samples, including 11 from the family *CAG-508* (*Clostridia* order *TANB77*, Supplementary Table 24). All putatively lost species were identified in at least one wild rodent host beyond *M. m. domesticus;* none overlapped with species lost based on faecal samples. All the species identified in four bacterial families were inferred to have been gained in the laboratory environment within caecal samples: *Tannerellaceae* (*n* = 7), *Akkermansiaceae* (*n* = 3), *Desulfovibrionaceae* (*n* = 3), *Enterococcaceae* (*n* = 3) and *Rikenellaceae* (*n* = 2) (Supplementary Table 25). The majority (97%) of species lost were members of the family *Lachnospiraceae* (*n* = 47) or *Oscillospiraceae* (*n* = 18) however these only represented ~4% of total species present from each of these families.

### *Muribaculaceae* and the rodent host

**Fig. 7:**
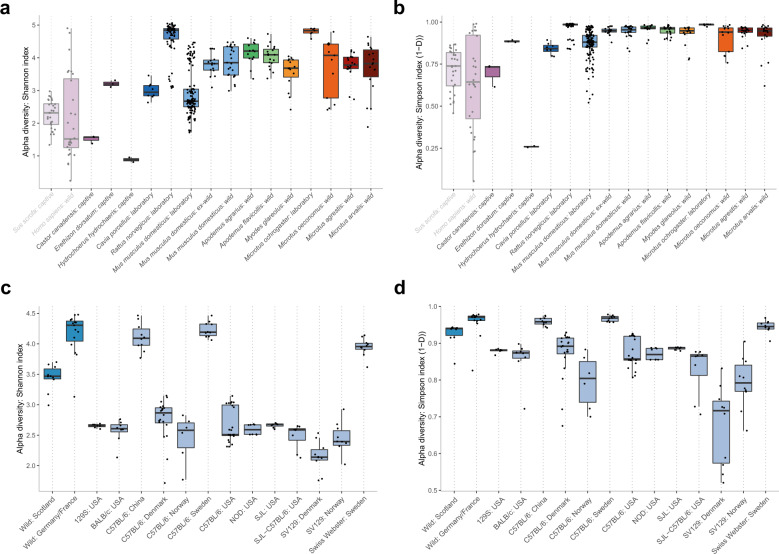
Laboratory mice exhibit reduced diversity within the family *Muribaculaceae*. Alpha diversity of (**a**, **b**) rodent hosts and (**c, d**) laboratory mouse strains measured using Shannon and Simpson (1-D) indices based on genome size scaled mapping counts. Significance determined using Dunn’s multiple comparison test with Benjamini-Hochberg adjustment—see Supplementary (Tables 26 and 27). Global analysis undertaken using Kruskal-Wallis test (*p* < 0.0001). Hosts represented by <5 samples excluded (*Castor canadensis*, *Erethizon dorsatum*, *Hydrochoerus hydrochaeris*). Analysis includes untreated faecal samples only.

**Fig. 8:**
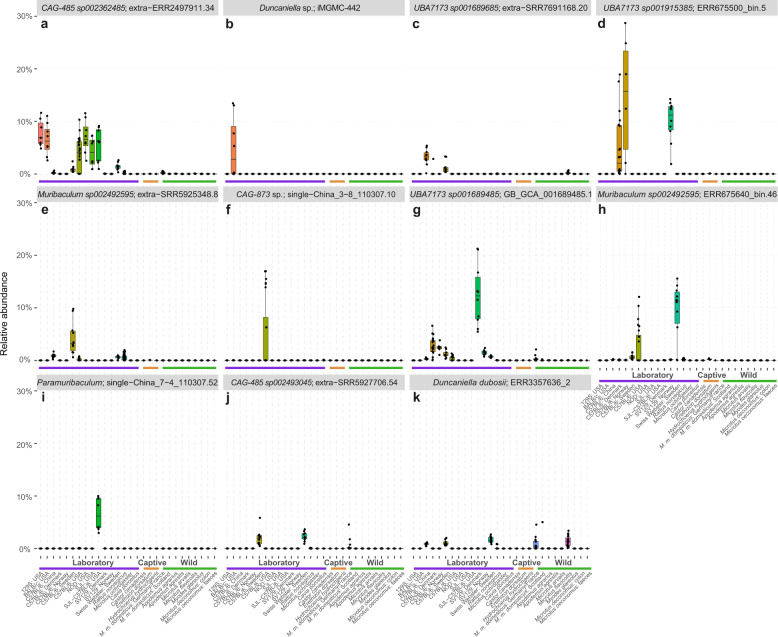
Outgrowths of members of the family *Muribaculaceae* are apparent between laboratory cohorts. Relative abundance within faecal and colon samples of the (**a–i**) species with highest abundance from each laboratory cohort and (**j-k**) highest abundance in wild mouse populations. Samples from untreated laboratory mice only are included.

The family *Muribaculaceae* belonging to the *Bacteroidota* phylum is frequently observed as fluctuating in abundance in studies of laboratory mice and can range from below detection to representing over half of the bacterial gut community [79–81] making it of interest for further analysis. While the abundance of the family in faecal samples from wild mice also varied substantially, it was typically lower than that of laboratory mice (mean relative abundance 11% in wild compared to 33% in laboratory mice). However, the abundance of *Muribaculaceae* in faeces/colon samples of other rodent hosts was mostly similar to or higher than that of laboratory mice, as previously observed based on 16S rRNA amplicon sequencing [18, 82], suggesting that this family is historically dominant in rodents (Supplementary Fig. 9). Despite the comparable abundance of *Muribaculaceae* in laboratory mice and wild rodents, the species diversity of the family was significantly reduced in the former (Fig. 7a, b, Supplementary Table 26) due to localised outgrowths of one or more *Muribaculaceae* species within laboratory cohorts (Fig. 8). The reduced diversity was apparent in most laboratory mouse cohorts, although some retained a level of diversity comparable to wild mice (Fig. 7c, d, Supplementary Table 27). The diversity of *Muribaculaceae* was also not reduced in all laboratory hosts, with laboratory rats and voles displaying increased diversity in comparison to most hosts, further emphasising the lack of a generic “laboratory” effect.

The increased abundance of *Muribaculaceae* in laboratory mouse faecal samples in comparison to wild mouse contrasts with the depleted status of this family in the analysed laboratory caecal samples. The family was almost entirely absent (average < 0.04%) in laboratory caecal and ileocecal samples (Supplementary Fig. 9c) [6, 7], however, comprised up to 30% of the caecal community in wild rodent hosts. The majority (99%) of *Muribaculaceae* species identified as present in wild mouse caecal samples were also present in laboratory mouse faeces indicating the depletion of the family in the laboratory mouse is not a consequence of absence of certain species from that environment (Supplementary Table 28). These species were also restored via re-wilding of laboratory mice, both via oral gavage of pregnant females [6] and embryonic transplant [7], confirming their depletion is also not a product of inability to colonise the caecum of laboratory mice. Furthermore, the family was present at higher abundance (average 46%, Supplementary Table 29) in caecal samples from additional studies [74, 75] indicating the depletion observed here is laboratory specific, as is observed across faecal samples (Supplementary Fig. 9b).

The pattern of *Muribaculaceae* species presence/absence across the dataset revealed distinct species clusters in some hosts, potentially a signal of host filtering (Supplementary Fig. 10). To assess whether clustering across the family was significant, we used two phylogenetic measures: mean phylogenetic distance (MPD), measuring the mean between species within a group, and mean nearest taxon distance (MNTD), measuring the mean distance to each species’ closest neighbour within a group. We observed a significant clustering signal (MNTD, indicative of tip clustering [83]) relative to a null model for all samples from the laboratory vole, *Microtus ochrogaster*, plus a subset of samples from other vole hosts, indicating *Muribaculaceae* species identified within these hosts are more closely related than expected by chance (Supplementary Fig. 11). This suggests that host specific characteristics such as diet or physiology may be influential in determining the range of *Muribaculaceae* species able to inhabit these hosts. Host specific clades did not clearly overlap with metabolic guild membership based on the abundance of defining carbohydrate-active enzymes of each guild: α-glucan guild (GH13), plant glycan guild (GH5, GH10, GH25, GH43 and GH51) and host glycan guild (GH20 and GH29) (Supplementary Fig. 10) [84]. Furthermore, no clear separation was apparent between host specific (specific to either an individual rodent host, host genus or host family) and non-host specific *Muribaculaceae* species based on multivariate analysis of the guild-defining enzyme profiles (sPLS-DA balanced error rate 0.48). We therefore undertook a comparison of CAZy profiles of *Muribaculaceae* species separating each of four host group pairs: wild and laboratory mice, wild and laboratory Murinae hosts, wild and laboratory voles and wild Murinae and vole hosts (Supplementary Fig. 12). Species identified in laboratory mice but not wild mice were enriched for enzymes involved in peptidoglycan biosynthesis (GT4, GT9, GT30, GT51), as well as enzymes with potential to metabolise host components such as blood group antigens, chondroitin and mucin (PL8, GH20, GH29, GH109, GH123) (Supplementary Tables 30-31). *Muribaculaceae* members specific to the laboratory host may therefore have greater potential to be inflammatory or invasive, consistent with a pathobiont role [85] and of particular interest given their capacity to reach high abundance (Fig. 8, Supplementary Table 31). Species identified in wild but not laboratory mice were enriched in a range of enzymes associated with degradation of plant components e.g., xylan and cellulose (CBM6, CBM9, CBM36, GH10, GH115, GH141) (Supplementary Tables 30–31), likely reflecting the more diverse diet of wild mice. A similar trend was observed amongst *Muribaculaceae* species identified in laboratory mice or rats but absent from wild Murinae hosts, as well as those found in laboratory voles but not detected in wild voles: enzymes involved in peptidoglycan synthesis and degradation were enriched in laboratory hosts and plant degradative enzymes were enriched in wild hosts (Supplementary Tables 30 and 32–33). *Muribaculaceae* species present in wild voles but not laboratory voles were also enriched for enzymes with potential to cleave fucose and sialic acid from mucin (GH33 and GH95), in contrast to the observed enrichment of mucin degrading potential within laboratory mice vs wild mice. Finally, comparison of species identified within either wild Murinae or wild vole hosts identified enrichment of a different complement of enzymes involved in plant degradation in each group, with mannan degradative enzymes enriched in Murinae hosts and pectate enzymes enriched in vole hosts. This may indicate differing dietary preferences between these rodents for plants with contrasting cell wall types, for example seeds are rich in mannan, whereas some fruits are rich in pectin (Supplementary Tables 30 and 34). Diet as a key driver of *Muribaculaceae* species abundance is consistent with sensitivity of the family to dietary interventions in the laboratory e.g., to high-fat diets [86, 87].

## Conclusions

Here we present a study of the rodent gut microbiome which extends our understanding beyond the laboratory mouse using a combination of wild, laboratory and captive individuals across 16 rodent hosts. We identified members of the bacterial families *Lachnospiraceae* and *Oscillospiraceae* to be the most broadly distributed across rodent hosts, however, no species were detected across all rodents within the dataset. Species from the family *Lachnospiraceae* were also the most numerous amongst bacterial species identified within a single rodent host, followed by the family *Muribaculaceae*. Comparison of laboratory mice with their wild ancestors revealed members of the family *Helicobacteraceae* were significantly depleted in laboratory mice, a finding that was revealed to be laboratory specific, consistent with targeted depletion. A number of species from the family *Muribaculaceae* were significantly enriched within laboratory mice, although the overall diversity of this family was reduced in most laboratory cohorts with only three (of 13) retaining comparable diversity to that of wild mice. Furthermore, *Muribaculaceae* species associated with laboratory rodents had higher predicted potential to be inflammatory or invasive relative to wild rodent *Muribaculaceae* species, which were instead enriched in plant degradative enzymes consistent with a more diverse diet. Members of several bacterial families with pathobiont potential also appear to have been gained within the laboratory environment, emphasising the importance of considering the origin of clinically important microbial taxa in model host organisms.

## Supplementary information

Supplementary figures

Supplementary tables

## Data Availability

Sequencing data generated in this study and MAGs assembled from these data are available via NCBI BioProject PRJNA725899. All assembled MAGs (including those generated from public data) are available via 10.5281/zenodo.5039598.
