# Supplementary material for: Effects of laboratory domestication on the rodent gut microbiome
Source: ISME Commun. 2021 Sep 17;1:49. doi: 10.1038/s43705-021-00053-9 (PMC9723573; doi:10.1038/s43705-021-00053-9)
Supplement: Supplementary file 1 — Supplementary figures [file 43705_2021_53_MOESM1_ESM.pdf]

Supplementary Figures

Effects of laboratory domestication on the rodent gut microbiome

Kate L. Bowerman[1], Sarah C. L. Knowles[2], Janette E. Bradley[3], Laima Baltrūnaitė[4], Michael D. J. Lynch[5], Kathryn M. Jones[6], Philip Hugenholtz[1]

[1]Australian Centre for Ecogenomics, School of Chemistry and Molecular Biosciences, The University of Queensland, Queensland, Australia.

[2]Department of Zoology, University of Oxford, Oxford, UK

[3]School of Life Sciences, University of Nottingham, Nottingham, UK

[4]Nature Research Centre, Akademijos Str. 2, Vilnius, Lithuania

[5]Department of Biology, University of Waterloo, Ontario, Canada

[6]Department of Biological Science, Florida State University, Florida, USA

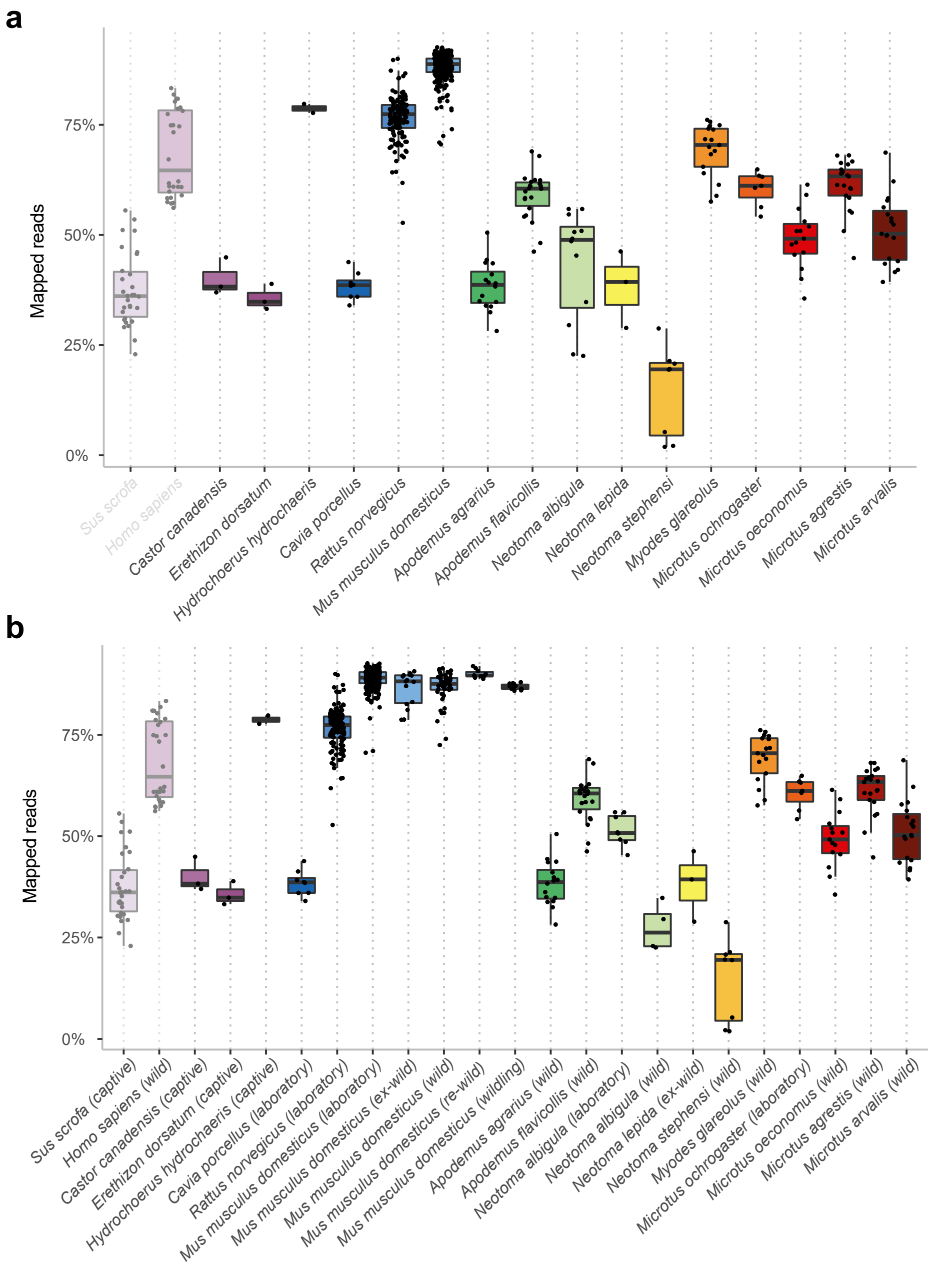

Supplementary Fig. 1. Read recruitment is biased toward model species. Read recruitment per sample for each **a** host and **b** host population type to the genome database. Boxes are drawn from the 25th to the 75th quantiles and centre line represents the median.

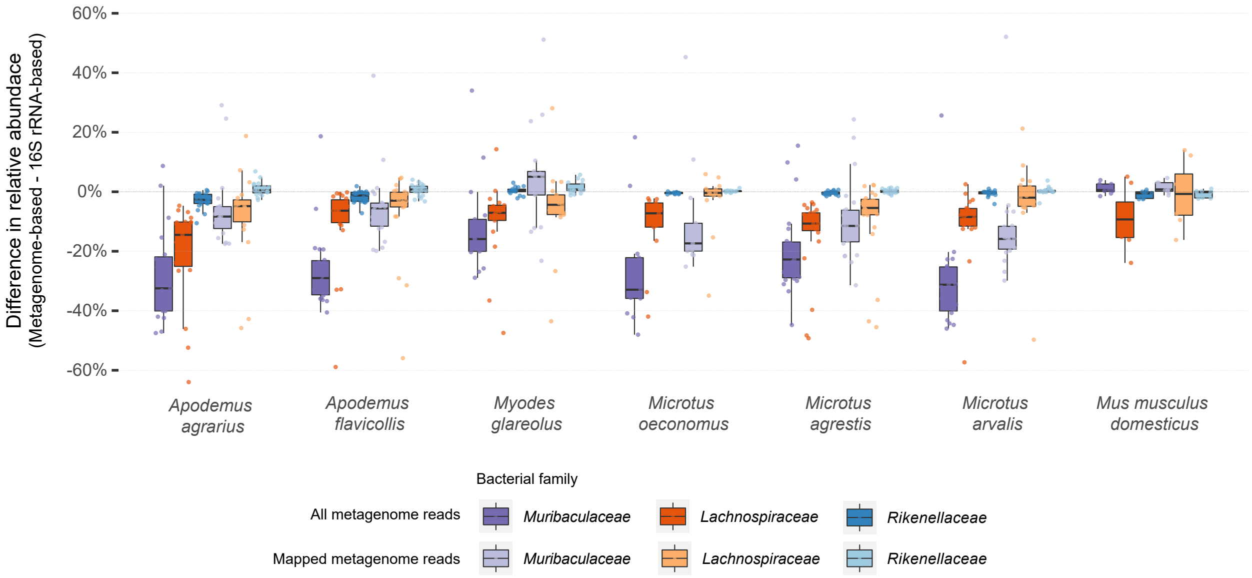

Supplementary Fig. 2. Metagenome-based relative abundance derived from mapped reads only closely resembles that from 16S rRNA gene amplicon-based analysis. Relative abundance of dominant bacterial families from metagenomic and 16S rRNA gene sequencing of the same samples using all metagenome reads in comparison to only reads recruited to the genome database i.e. excluding unmapped reads.

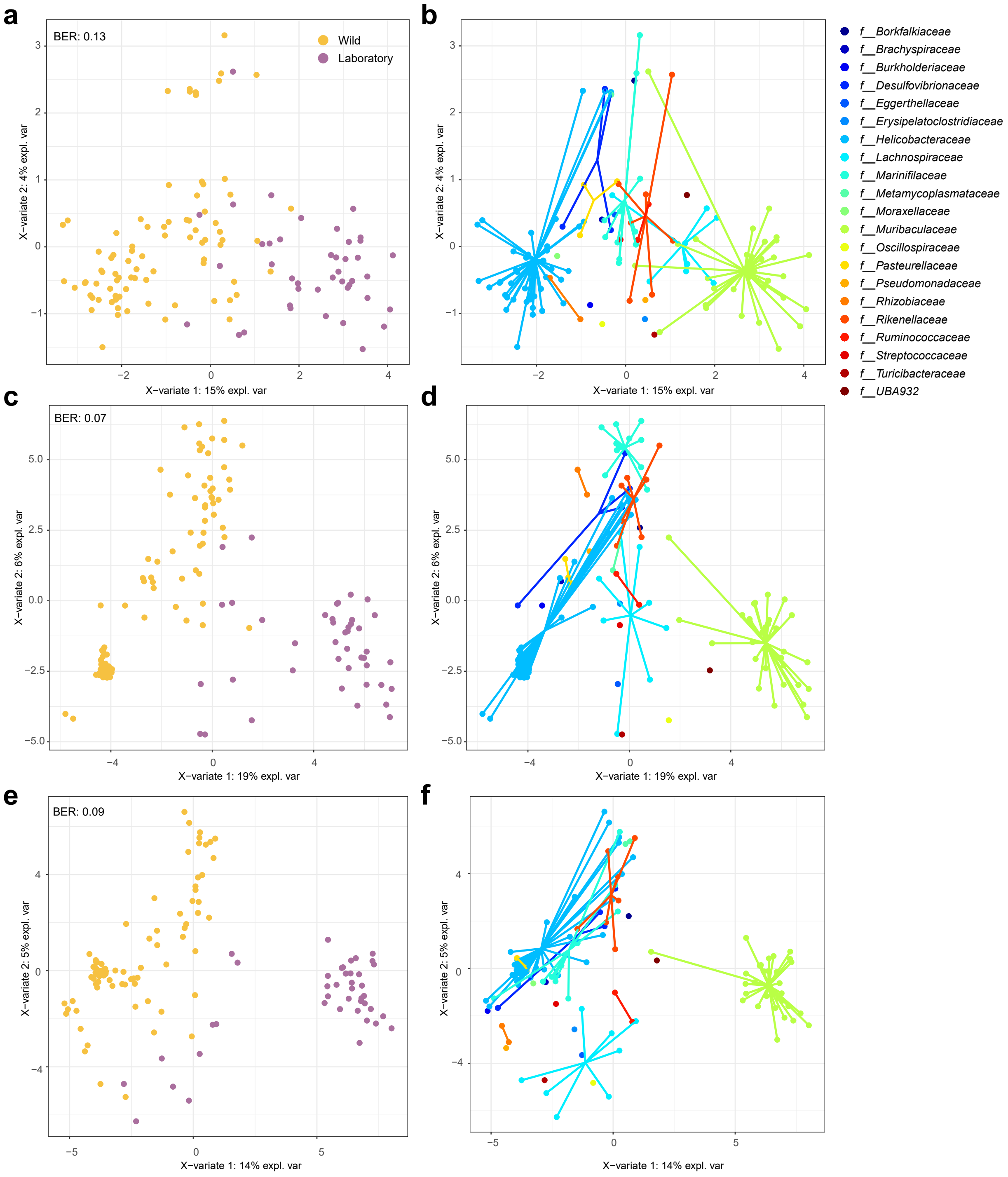

Supplementary Fig. 3. Functional annotations of *Muribaculaceae* and *Helicobacteraceae* drive separation between laboratory and wild mouse associated species. sPLS-DA based on **a, b** CAZy, **c, d** KEGG and **e, f** Pfam annotation profiles of genomes enriched in either laboratory or wild mice. Colouring indicates either **a, c, e** mouse group in which genome enriched or **b, d, f** bacterial family of genome. Annotations filtered for those present in ≥10% of the discriminatory genomes. BER indicates balanced error rate of sPLS-DA.

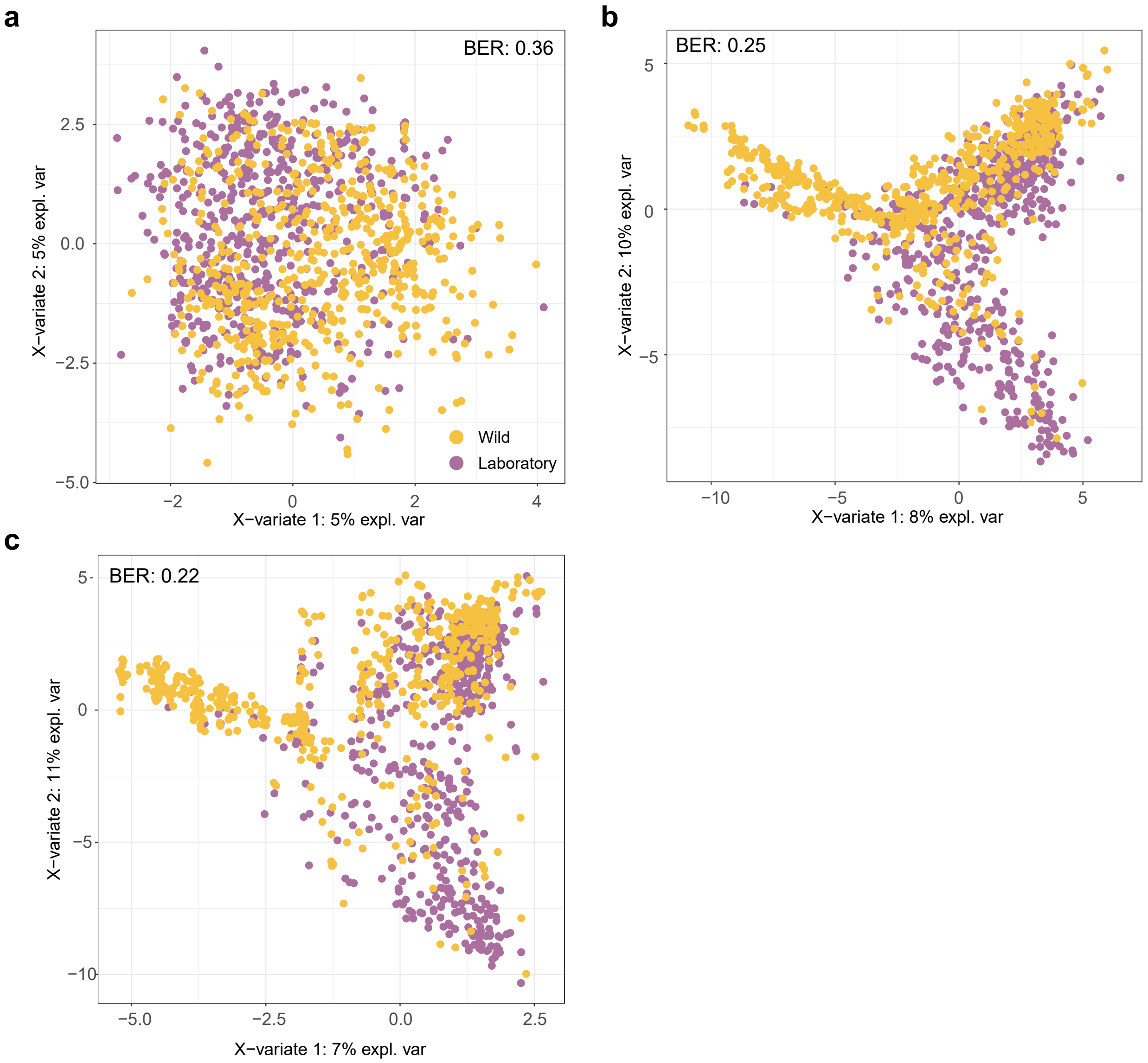

Supplementary Fig. 4. Functional annotations of genomes enriched in laboratory or wild *Microtus* voles. sPLS-DA based on **a** CAZy, **b** KEGG and **c** Pfam annotation profiles of genomes enriched in either laboratory or wild Microtus voles. Colouring indicates enrichment in either laboratory (purple) or wild (yellow) hosts. Annotations filtered for those present in ≥10% of the discriminatory genomes. BER indicates balanced error rate of sPLS-DA.

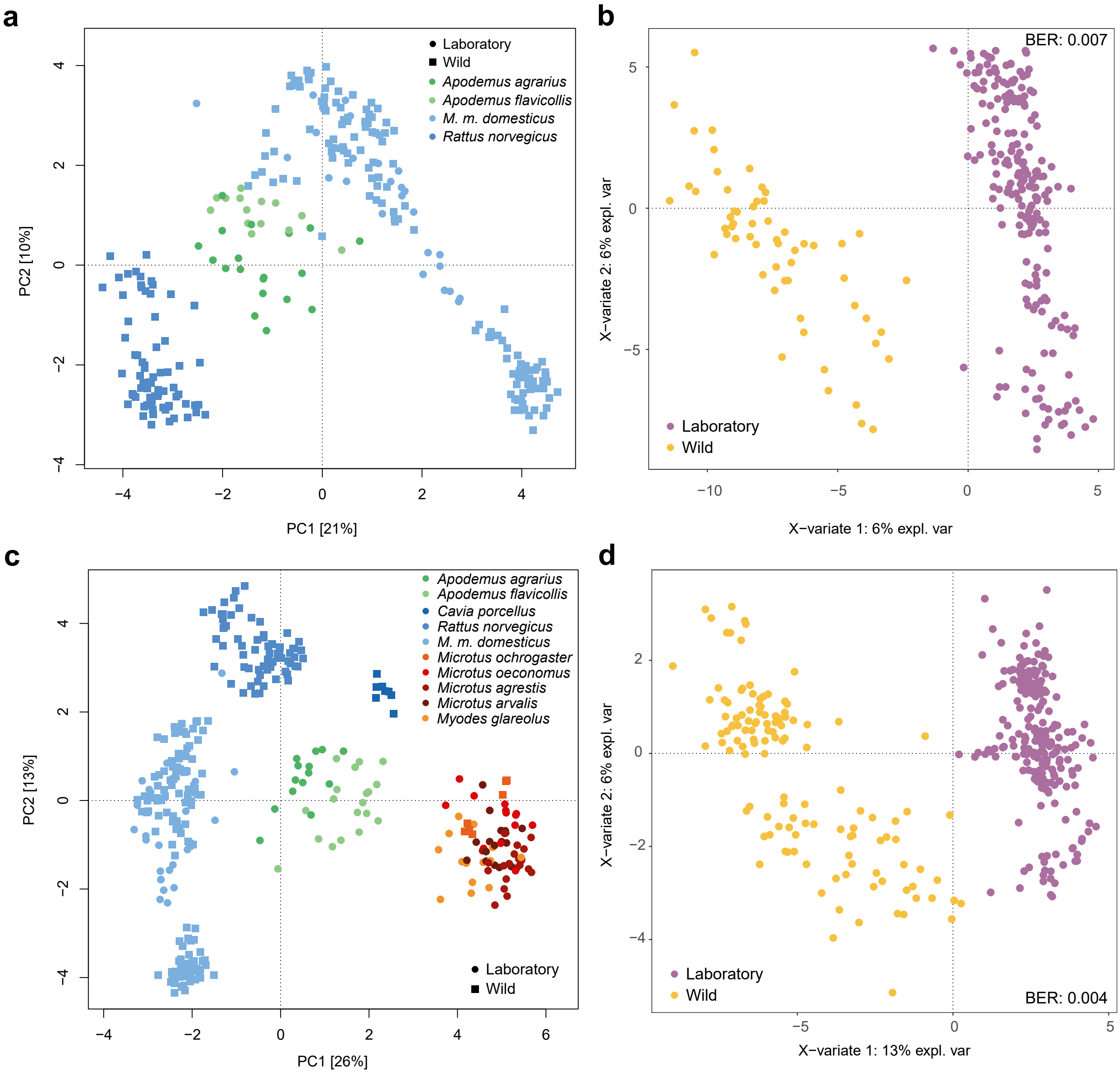

Supplementary Fig. 5. Extended comparison of faecal samples from laboratory and wild hosts. PCA of **a** Murinae and **b** all laboratory and wild hosts, coloured by host. sPLS-DA based on **b** Murinae and **d** all laboratory and wild faecal samples, coloured by source. BER indicates balanced error rate of sPLS-DA. Analysis based on read mapping counts (CLR-transformed) to genome database filtered to include genomes recruiting ≥500 reads across ≥0.01 of the genome in ≥1 sample.

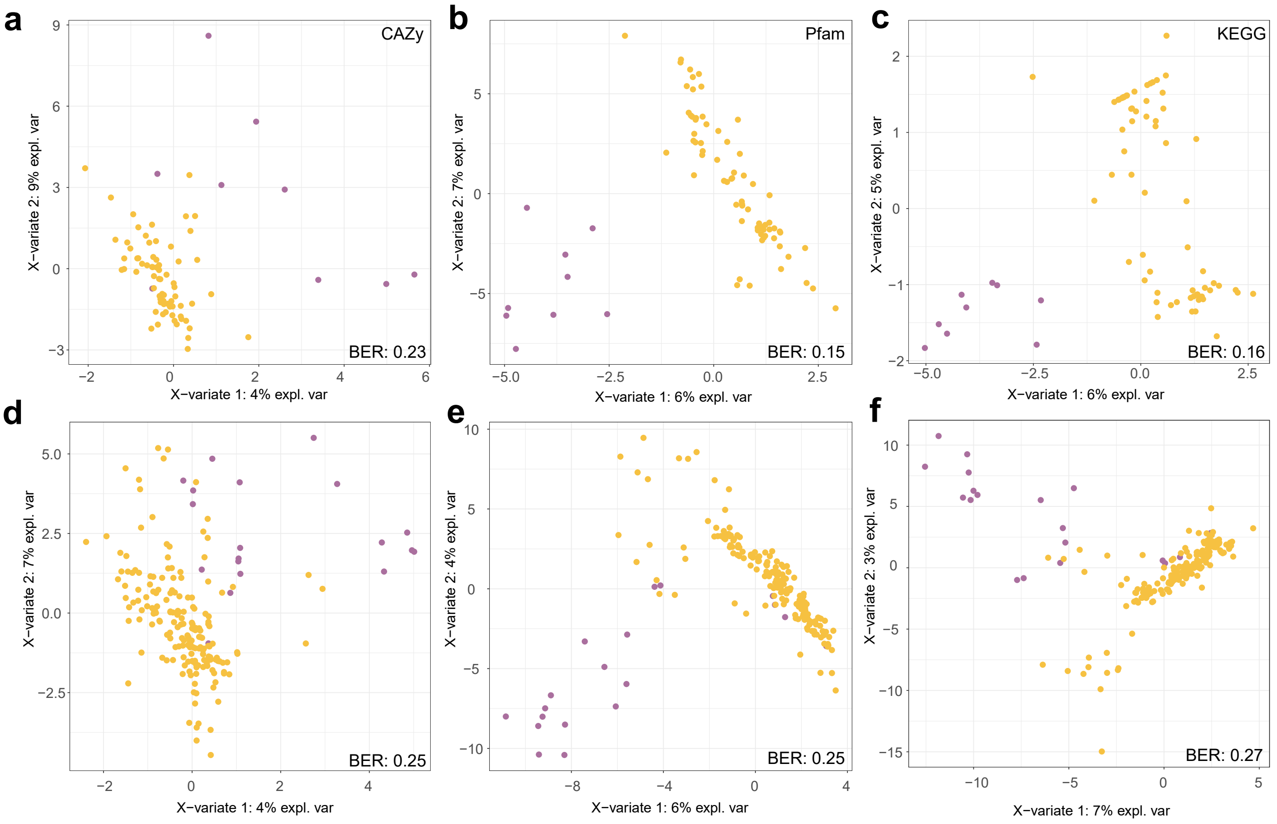

Supplementary Fig. 6. Some distinction is evident between functional annotations of genomes enriched in faecal community of groups of laboratory or wild hosts. sPLS-DA based on **a,d** CAZy, **b,e** Pfam and **c,f** KEGG annotation profiles of genomes enriched in either laboratory or wild **a-c** Murinae or **d-f** all hosts. Colouring indicates enrichment in either laboratory (purple) or wild (yellow) hosts. Annotations filtered for those present in **a-c** ≥5 or **d-f** ≥8 of the discriminatory genomes. BER indicates balanced error rate of sPLS-DA.

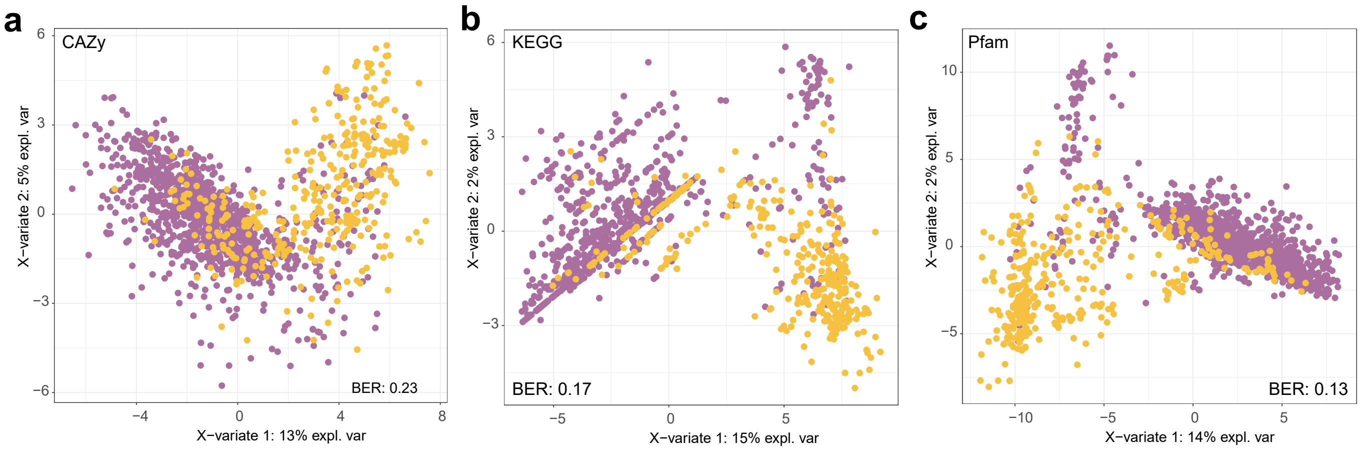

Supplementary Fig. 7. Some distinction is evident between functional annotations of genomes enriched in cecal community of laboratory or wild hosts. sPLS-DA based on **a** CAZy, **b** Pfam and **c** KEGG annotation profiles of genomes enriched in either laboratory or wild hosts. Colouring indicates enrichment in either laboratory (purple) or wild (yellow) hosts. Annotations filtered for those present in ≥150 of the discriminatory genomes. BER indicates balanced error rate of sPLS-DA.

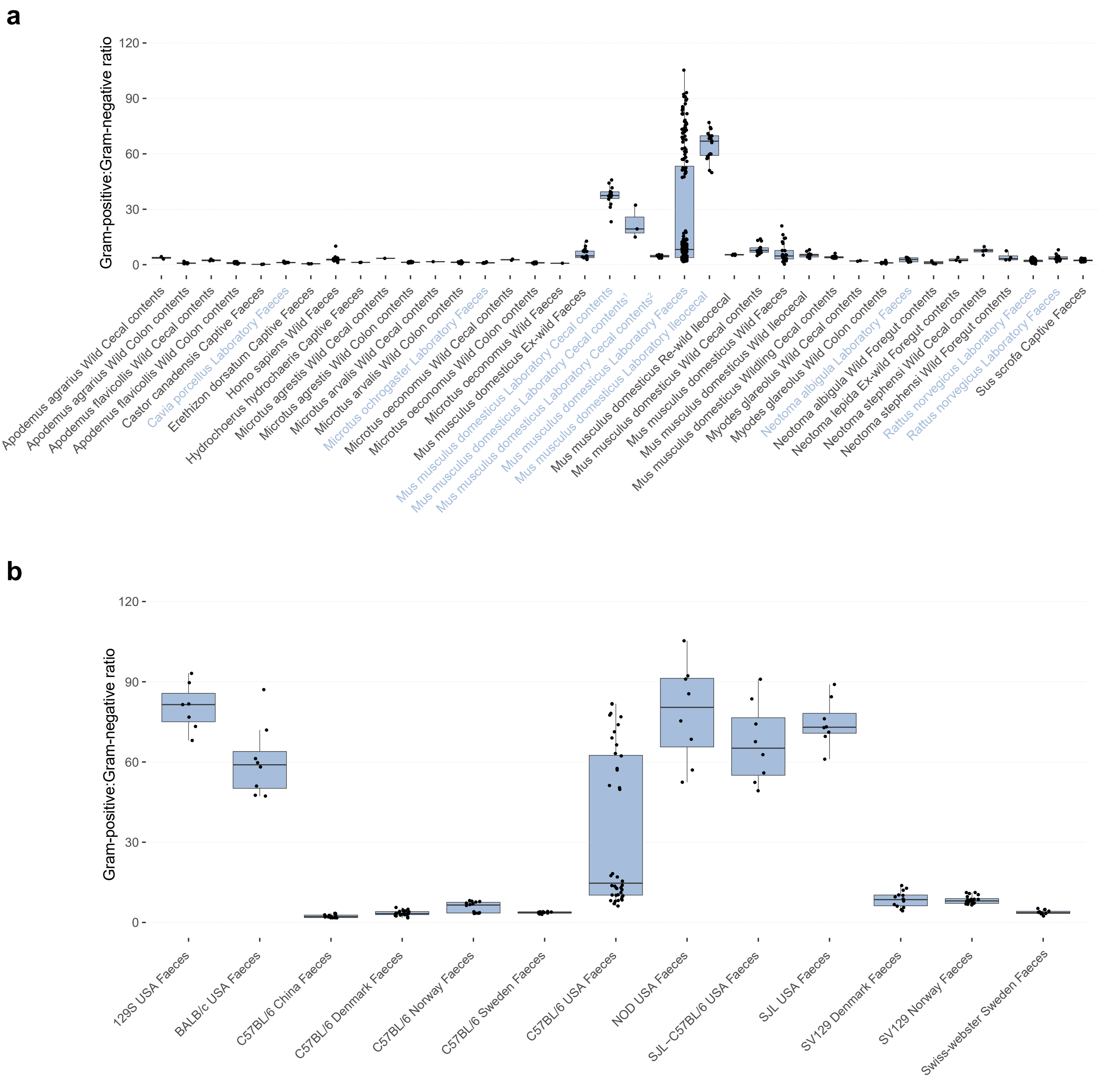

Supplementary Fig. 8. Gram-positive:Gram-negative ratio is inflated in faecal and cecal samples of some laboratory mice. Ratio of Gram-positive:Gram-negative bacterial species in **a** all samples from rodent hosts and **b** faecal samples from laboratory mice. Gram status determined by Pfam annotations identified per genome. Includes genomes within each host group meeting a read recruitment threshold of ≥500 reads across ≥0.01 of the genome in ≥1 sample for that specific group.

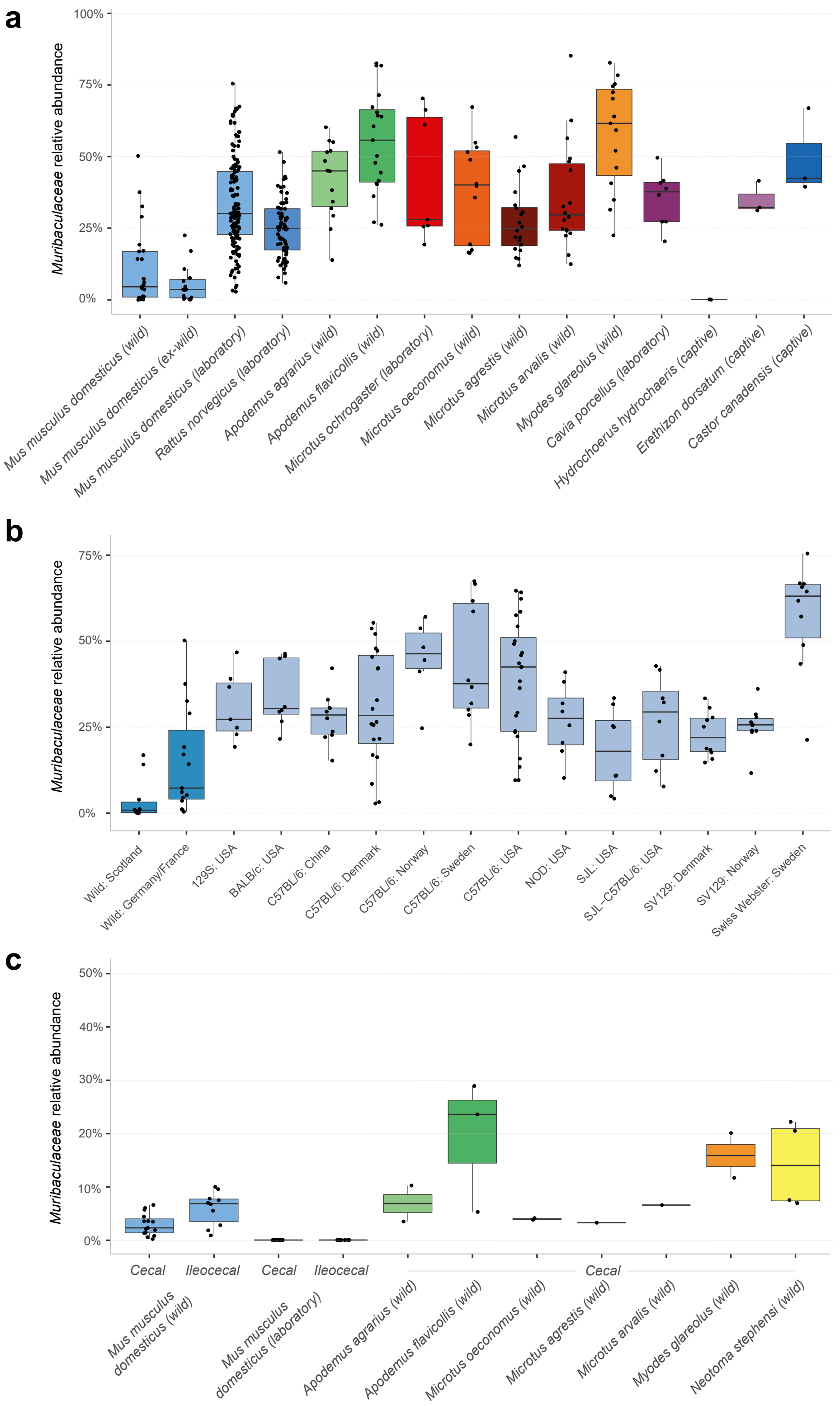

Supplementary Fig. 9. The family *Muribaculaceae* is dominant within the faecal community of multiple rodent hosts. Relative abundance of the family *Muribaculaceae* within **a** faecal samples of rodent hosts, **b** faecal samples from laboratory mice (by strain and laboratory) and **c** cecal and ileocecal samples of rodent hosts.

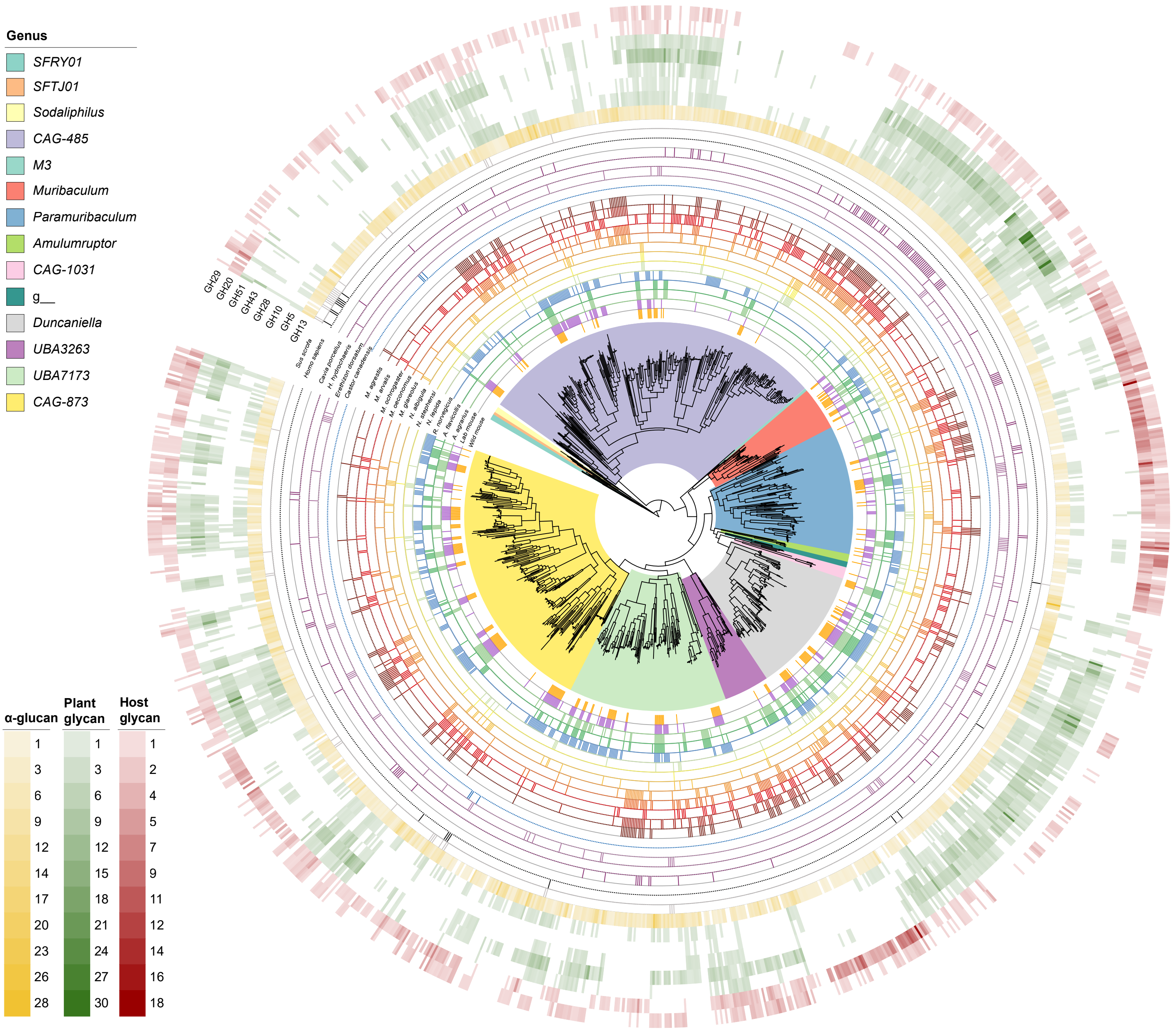

Supplementary Fig. 10. Distribution of the family *Muribaculaceae* across the rodent gut microbiome. Maximum likelihood tree from Fig. 1 pruned to *Muribaculaceae* (1,210 genomes). Inner rings show presence of each species in a given rodent host based on recruiting ≥500 reads across ≥0.01 of the genome in ≥1 sample (16 rings), followed by outgroup species, *Homo sapiens* and *Sus scrofa*. Heatmap displays count of CAZy annotations per genome for enzymes characteristic of trophic guilds described in Ormerod KL, Wood DLA et al. 2016. Coloured ranges define genera.

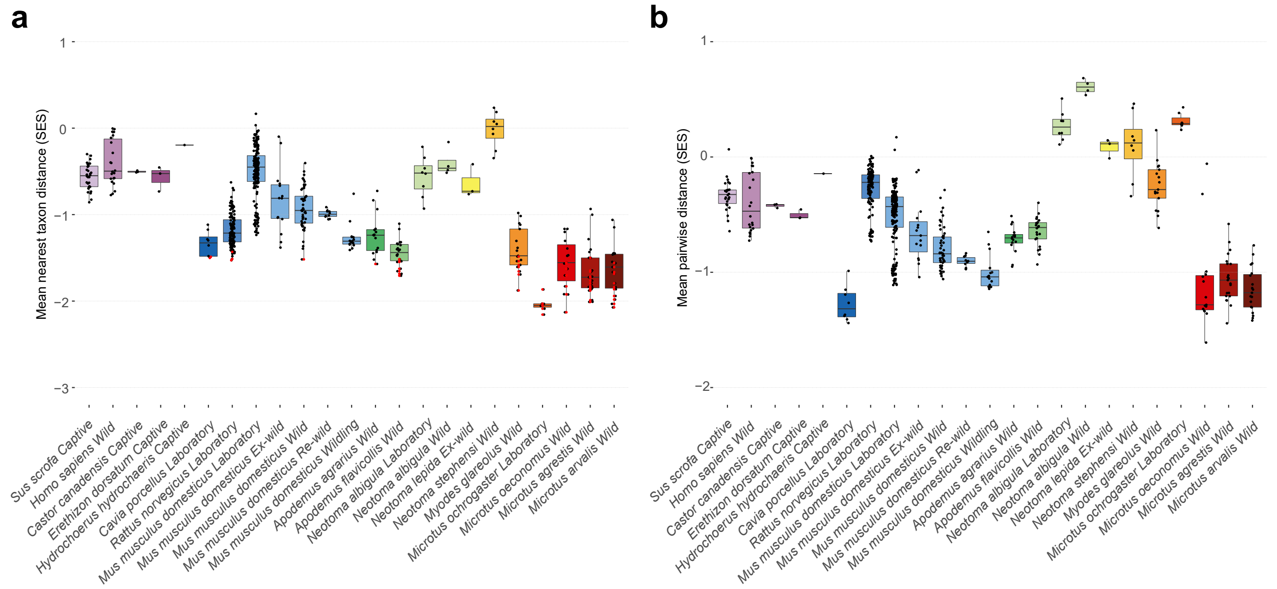

Supplementary Fig. 11. Phylogenetic clustering within the family *Muribaculaceae* across rodent gut microbiome samples. Standardized **a** mean nearest taxon distance and **b** mean pairwise distance scores per rodent host species population type. Red points indicate significant deviation from an independent swap null model maintaining species occurrence frequency and sample species richness. Boxes are drawn from the 25th to the 75th quantiles and centre line represents the median.

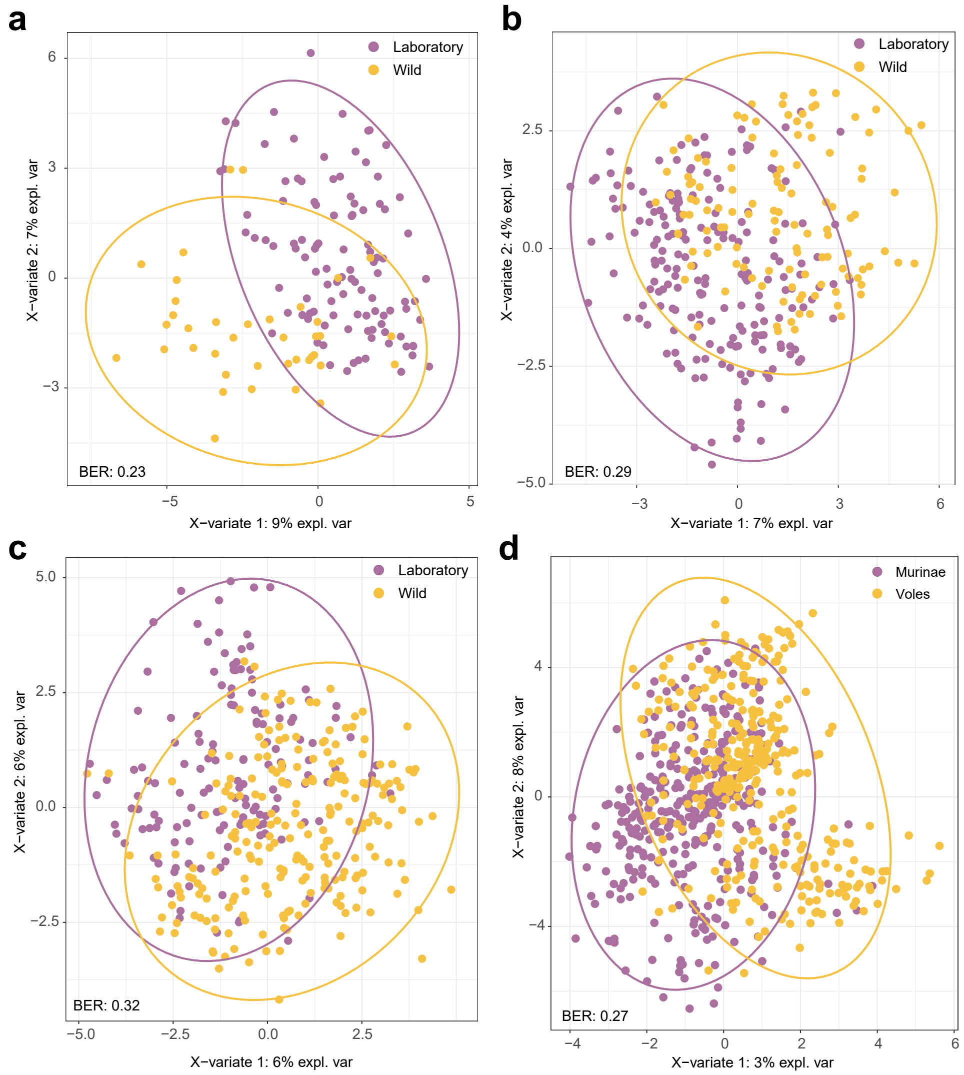

Supplementary Fig. 12. Predicted carbohydrate interaction profile of *Muribaculaceae* species within rodent host groups. sPLS-DA based on CAZy annotation profiles of genomes present in either **a** laboratory or wild mice, **b** laboratory or wild Murinae hosts, **c** laboratory or wild voles and **d** wild Murinae or vole hosts. Colouring indicates presence in either group. Annotations filtered for those present in ≥10 of the discriminatory genomes. BER indicates balanced error rate of sPLS-DA.
